# Chemical recognition of fruit ripeness in spider monkeys (*Ateles geoffroyi*)

**DOI:** 10.1038/srep14895

**Published:** 2015-10-06

**Authors:** Omer Nevo, Rosa Orts Garri, Laura Teresa Hernandez Salazar, Stefan Schulz, Eckhard W. Heymann, Manfred Ayasse, Matthias Laska

**Affiliations:** 1 Behavioral Ecology & Sociobiology Unit, German Primate Center, Kellnerweg 4, 37077 Göttingen, Germany; 2 Department of Sociobiology/Anthropology, Johann-Friedrich-Blumenbach Institute for Zoology and Anthropology, Georg-August University of Göttingen, Kellnerweg 6, 37077 Göttingen, Germany; 3 Institute of Evolutionary Ecology and Conservation Genomics, University of Ulm, Helmhotztr. 10-1, Containerdorf, 89081 Ulm, Germany; 4 IFM Biology, Linköping University, SE-58183 Linköping, Sweden; 5 Institute of Neuroethology, University of Veracruzana, Calle y No. Dr. Luis Castelazo s/n, Col. Industrial Animas, C.P. 91190, Ciudad, Xalapa, Ver., México; 6 Institute of Organic Chemistry, Technical University of Braunschweig, Hagenring 30, 38106 Braunschweig, Germany

## Abstract

Primates are now known to possess well-developed olfactory sensitivity and discrimination capacities that can play a substantial role in many aspects of their interaction with conspecifics and the environment. Several studies have demonstrated that olfactory cues may be useful in fruit selection. Here, using a conditioning paradigm, we show that captive spider monkeys (*Ateles geoffroyi*) display high olfactory discrimination performance between synthetic odor mixtures mimicking ripe and unripe fruits of two wild, primate-consumed, Neotropical plant species. Further, we show that spider monkeys are able to discriminate the odor of ripe fruits from odors that simulate unripe fruits that become increasingly similar to that of ripe ones. These results suggest that the ability of spider monkeys to identify ripe fruits may not depend on the presence of any individual compound that mark fruit ripeness. Further, the results demonstrate that spider monkeys are able to identify ripe fruits even when the odor signal is accompanied by a substantial degree of noise.

Primates have traditionally been considered as primarily visually-oriented animals with a poorly developed sense of smell^1^,^2^. This view was mainly, if not exclusively, based on an interpretation of neuroanatomical and – more recently – genetic findings and not on behavioral or physiological evidence^3^. Meanwhile, an increasing number of studies now suggest that olfaction may play a significant role in regulating a wide variety of primate behaviors^4^,^5^ and that the olfactory sensitivity and discrimination capacities of both human and nonhuman primates are not generally inferior to that of nonprimate species believed to have a keen sense of smell^6^.

A high olfactory sensitivity and well-developed olfactory discrimination capabilities have been reported in several primate species of different lineages^3^,^6^,^7^,^8^. These olfactory capabilities have been shown to be particularly tuned to detection, discrimination and identification of compounds common in fruits^8^,^9^,^10^,^11^,^12^. Additionally, behavioral studies from captivity and the wild demonstrated that non-human primates can rely on olfactory cues in fruit selection tasks^13^,^14^.

While psychophysical studies quantified the olfactory sensitivity for and discrimination performance of primates with compounds that are potentially relevant for feeding on fruits^8^,^10^, they all used monomolecular stimuli. Yet fruit odor is composed of complex mixtures of odorants^15^,^16^ and in natural fruit-selection primates need to identify complex odor signatures of ripe fruits and be able to discriminate them from odor profiles of unripe fruits. Thus, although these studies showed a potential to rely on olfactory cues in frugivory, they could not demonstrate whether and how high olfactory sensitivity and discrimination capacity translate into success in food acquisition tasks mimicking a real-life situation. On the other hand, most behavioral studies that employed more ecologically-realistic approaches used chemically undefined stimuli^17^,^18^,^19^ or chemically known stimuli of domesticated fruit species^20^ whose odor profiles may have been enhanced through artificial selection and are therefore not representative of the challenges primates face when selecting fruits in the wild. So, the connection between primate olfactory physiology and feeding ecology is not yet fully established. First, the ability to discriminate between complex odor mixtures that mimic odors of wild ripe and unripe fruits has not been experimentally demonstrated. Second, it is unknown whether sensitivity to, or discrimination of, any particular compound or compound family disproportionally increases their capability to identify ripe fruits.

A recent study (Nevo *et al.*, unpublished data) provided detailed analyses of the chemical profiles of the fruit odors of two Neotropical plant species whose seeds are dispersed primarily by primates. By comparison to patterns of odor emission in fruits whose main seed-dispersal vectors are birds, which are demonstrated to be less olfactory-, and more visually, dependent, it was hypothesized that odors of ripe fruits consumed by primates are not merely a cue that primates can potentially exploit, but an evolved signal whose function is to facilitate the communication between seed-dispersing primates and plants. More specifically, it was suggested that since primates and other, extant or extinct, frugivores tend to use olfactory cues in fruit selection^13^, fruits that rely on their seed-dispersal services have evolved to emit an odor which is unique to the ripe phase, i.e. significantly different from the odor of unripe fruits of the same species. This was hypothesized to increase the ability of primates and other frugivores to identify ripe fruits when selecting between ripe and unripe fruits in a feeding tree and hence their foraging efficiency. In turn, this should increase the overall attractiveness of a fruit and allow the plant to outcompete con- and heterospecifics in attracting dispersal vectors. However, without bioassays that test the ability of primates to detect the odors and discriminate them from the odors of unripe fruits, this claim remains tentative.

The current study attempts to build upon these chemical analyses of odors emitted by primate-consumed fruits and takes a step forward in connecting primate olfactory physiology and feeding ecology. Nevo *et al.* provide chemical characterizations of the odors of ripe and unripe fruits of two Neotropical plant species, *Couma macrocarpa* (Apocynaceae) and *Leonia cymosa* (Violaceae). Both plant species provide indehiscent fruits (i.e. whose peel does not open upon maturation) with a soft, leathery peel that acquires a yellow color when ripening. Analyses of the chemical profiles revealed that in one species, *C. macrocarpa*, peel odor (i.e. odor of the intact fruits) profiles of ripe and unripe fruits are strong, rich and distinct (i.e. different between ripe and unripe fruits). In contrast, intact ripe and unripe *L. cymosa* fruits bear weak and similar odors, whereas the pulp (open fruits) is very odorous and its composition differs significantly between ripe and unripe fruits. Thus, it was predicted that in *C. macrocarpa* the odor of intact fruits would be sufficient to inform primates that an individual fruit is ripe while in *L. cymosa* primates must first manipulate the fruits and expose the pulp, and only in this phase receive the signal that the fruit is ripe.

The goal of the current study was to assess whether the odor profiles of fruits of these two species indeed have the potential to mediate the interaction between plants and primates by signaling ripeness at the fruit selection phase, and to examine whether this ability depends on few compounds or compound classes that characterize the odors of ripe fruits. Using five captive black-handed spider monkeys (*Ateles geoffroyi*) as a model system, we addressed the following questions:

1. Can spider monkeys discriminate between odor profiles of ripe and unripe fruits (*C. macrocarpa*: peel odor, *L. cymosa*: peel and pulp odor)?
2. Does the ability to discriminate between odor profiles of ripe and unripe fruits of a certain plant species depend on one or a few compounds or compound classes that mark ripe fruits? Are odor profiles of unripe fruits that are more similar to the odor of ripe fruits with regards to these compounds more difficult to discriminate from the odor of ripe fruits?

To address these questions, we employed an olfactory conditioning paradigm^21^. We used synthetic odor mixtures that mimicked the odors of ripe and unripe fruits (peel odor in *C. macrocarpa* and both peel and pulp odors in *L. cymosa*). We first trained the monkeys to identify the odor of ripe fruits and associate it with a food reward. Then, to address the first question, we tested the ability of spider monkeys to discriminate between the odors of ripe and unripe fruits of each species in a given condition (intact or open) using the full odor mixtures (i.e. those mimicking the natural odors of ripe and unripe fruits, respectively, as closely as possible). To address the second question, we employed a series of similar experiments in which we tested the ability of spider monkeys to discriminate between the odor of *ripe* fruits and the odor of “partially ripe” fruits. Partially ripe fruits were odor mixtures similar to the odor of *unripe fruits* which were manipulated to resemble the odor of ripe fruits with regards to one or more compounds (i.e. the concentration of compounds was manipulated so that one or more compounds in the odor of unripe fruits were matched to their respective concentration in the odor of ripe fruits) (Table 1, 2, 3). Thus, in each of these experiments, the odor of unripe fruits became more similar to the odor of ripe fruits with regards to one or more compound while all the other odorants in the mixture remained at concentrations appropriate for unripe fruits. The question was whether this would decrease the monkeys’ ability to discriminate between the odors.

## Results

### *Couma macrocarpa* – peel odor (intact fruits)

**Figure 1:**
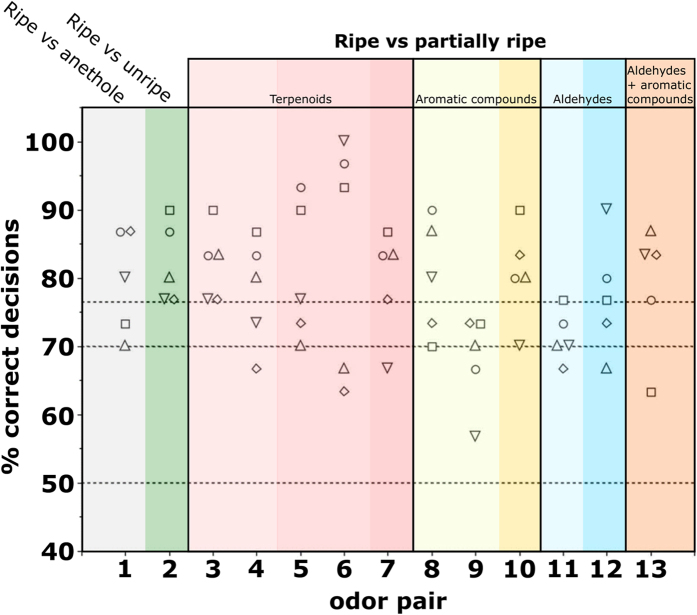
Performance of five spider monkeys in discriminating between the odor of ripe intact fruits of *Couma macrocarpa* and odor mixtures mimicking different degrees of ripeness of this fruit. Each data point represents the percentage of correct decisions per odor pair and animal. Horizontal lines indicate chance level at 50%, and criterion levels at 70% (corresponding to p < 0.05 in a binomial test; see methods) and at 76.7% (corresponding to p < 0.01). The numbers and composition of odor pairs are given in Table 1. Anethole (odor pair 1) served as a monomolecular training stimulus. Ripe vs unripe (odor pair 2) corresponds to question 1 from the introduction. Odor pairs 3–13 correspond to question 2. Colors in odor pairs 3–13 mark different odorant categories and darker shades within them (left to right) indicate increasingly ripe odor mixtures within these categories.

Figure 1 shows the performance of the five spider monkeys in the training phase (Fig. 1, odor pair 1) and in discriminating between the odor of intact ripe and unripe fruits of *Couma macrocarpa* (Q1, odor pair 2) and odors mimicking different degrees of ripeness of this fruit (Q2, odor pairs 3–13). With all 13 odor pairs, either all five animals (6 cases), or at least the majority of animals (7 cases) scored ≥70.0% correct decisions (corresponding to p < 0.05 in a binomial test; see methods) and therefore succeeded in discriminating between the stimuli above chance level. With 11 of the 13 odor pairs, the majority of animals even scored ≥76.7% correct decisions (corresponding to p < 0.01). Thus, the spider monkeys were clearly able to distinguish between odors of intact ripe and unripe fruits (Q1) and between ripe fruits and partially ripe fruits (Q2).

Discrimination performance did not differ between treatments (Friedman’s test: n = 5(11), χ^2^ = 14.59, p = 0.2), implying that none of the odor mixtures, including partially ripe odor mixtures that were more similar to the fully-ripe odor, were more difficult to discriminate compared to other odor mixtures.

### *Leonia cymosa* – peel odor (intact fruits)

Discrimination performance between odor profiles of ripe and unripe intact *Leonia cymosa* odors was overall very low. Mean success rates of 4 out of 5 spider monkeys was lower than 70% (mean: 58%) and thus not different from chance whereas one individual achieved 80% success. Success rates were equally low in the training phase (ripe fruits vs anethole) (mean: 59%). So, as a group, the monkeys showed difficulties in identifying the odor of ripe intact *L. cymosa* fruits and as a result could not recognize ripe fruits based on their odor in the intact condition. To exclude the possibility that the inability to discriminate the odor of ripe intact *L. cymosa* fruits from anethole or from the odor of intact unripe fruits derives from the inability to detect the odor, we tested the discrimination capacity from water. Success rates of all five individuals were equal to, or higher than, 76.7% (mean: 80%).

### *Leonia cymosa* – pulp odor (open fruits)

**Figure 2:**
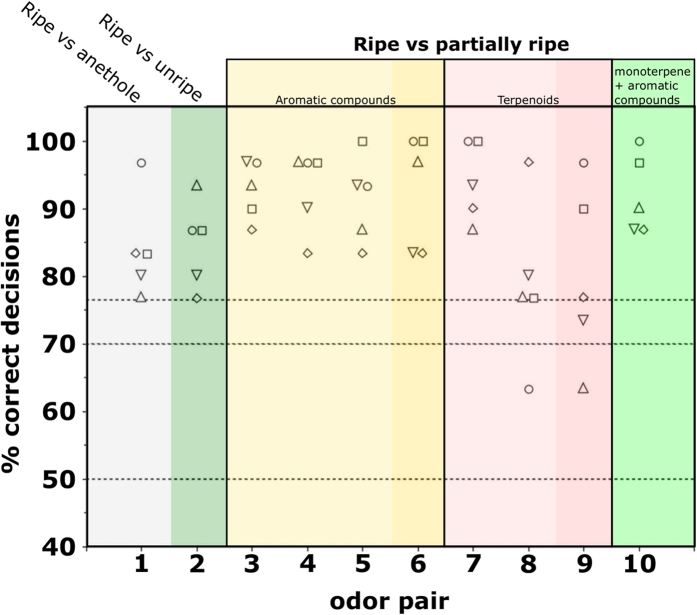
Performance of five spider monkeys in discriminating between the odor of ripe open fruits of *Leonia cymosa* and odor mixtures mimicking different degrees of ripeness of this fruit. Each data point represents the percentage of correct decisions per odor pair and animal. Horizontal lines indicate chance level at 50%, and criterion levels at 70% (corresponding to p < 0.05 in a binomial test; see methods) and at 76.7% (corresponding to p < 0.01). The numbers and composition of odor pairs are given in Table 3. Anethole (odor pair 1) served as a monomolecular training stimulus. Ripe vs unripe (odor pair 2) corresponds to question 1 from the introduction. Odor pairs 3–10 correspond to question 2. Colors in odor pairs 3–10 mark different odorant categories and darker shades within them (left to right) indicate increasingly ripe odor mixtures within these categories.

Figure 2 shows the performance of the five spider monkeys in the training phase (Fig. 1, odor pair 1) and in discriminating between the odor of open ripe and unripe fruits of *Leonia cymosa* (Q1, odor pair 2) and odors mimicking different degrees of ripeness of this fruit (Q2, odor pairs 3–10). With all 10 odor pairs, either all five animals, or at least the majority of animals succeeded in discriminating between the stimuli above chance level (p < 0.05). With 8 of the 10 odor pairs, all five animals even scored ≥76.7% correct decisions (corresponding to p < 0.01). Thus, the monkeys readily discriminated between odors of open ripe and unripe fruits and between the odor of ripe fruits and partially ripe fruits.

Differences in discrimination performance between treatments approached significance (Friedman’s test: n = 5(8), χ^2^ = 14.73, p = 0.065) but subsequent post-hoc analyses (pair-wise Wilcoxon Signed-Rank tests followed by the Bonferroni correction for multiple testing) revealed that discrimination performance in all tasks was statistically indistinguishable (all pairwise comparisons: adjusted p = 1). Thus, similar to intact *C. macrocarpa* fruits, none of the odor mixtures, including partially ripe odor mixtures that were more similar to the fully-ripe odor, were more difficult to discriminate compared to other odor mixtures.

## Discussion

The first question we addressed was whether spider monkeys can discriminate between the odors of ripe and unripe fruits. The results were positive in both plant species: in *C. macrocarpa* the animals successfully discriminated between the odors of intact (peel odor) ripe and unripe fruits (Fig. 1, odor pair 2); in *L. cymosa* they failed to do so, but could readily discriminate between the odors of open ripe and unripe fruits (pulp odor) (Fig. 2, odor pair 2). These results suggest that spider monkeys can rely on fruit odor for identification of ripe *C. macrocarpa* and *L. cymosa* fruits in the wild: during the food-selection process individuals sample the odors of ripe and unripe fruits. In an unordered series of visual, olfactory and tactile examination^22^, they are exposed to the odor of both the intact (peel odor) or open (pulp odor) fruit. As a result, they learn to associate the odors of ripe fruits with a reward, in a process that is similar to the conditioning paradigm employed here. Thus, over time, the ability to discriminate between odors of ripe and unripe fruits of these species is likely to translate into the ability to assess the fruits’ ripeness based solely on their odor.

These results are in line with the hypothesis that fruit odor in *C. macrocarpa* and *L. cymosa* is an evolved signal to seed-dispersing primates and/or other contemporary or extinct frugivores. Nevo *et al.* showed that the emission of a unique odor at ripeness, either of the intact or open fruits, characterizes plants whose seeds are dispersed by primates and not by birds. Our results confirm that spider monkeys have the ability to discriminate between the odors of ripe and unripe fruits and thus the potential to use fruit odor to identify ripe fruits. While alternative explanations cannot at this point be ruled out, these results indicate that selection by monkeys and other frugivores may have driven an evolution of unique odor at ripeness in fruit species whose seeds they disperse.

The second question we addressed was whether the ability of spider monkeys to discriminate between odors of ripe and unripe fruits depends on one or perhaps a few odorants or odorant classes which may be indicative of ripeness. Using an odor mixture that did not mimic a natural stimulus, a previous study has found that discrimination performance in squirrel monkeys (*Saimiri sciureus*) decreases when odor mixtures become increasingly similar and that some odorants disproportionally contribute to their ability to do so^23^. Here, using stimuli that mimicked natural fruit odors, only minor differences in the performance of the spider monkeys between the different odor pairs was observed and none was statistically significant. For example, in intact *C. macrocarpa*, adding methyl salicylate (Fig. 1, odor pair 9) to the odor of unripe fruits yielded a slight, but statistically insignificant, decrease in the spider monkeys’ discrimination performance. However, when adding all relevant aromatic compounds (Fig. 1, odor pair 10: methyl salicylate, ethyl salicylate, *p*-cymene), which was expected to yield an odor mixture that resembles the odor of ripe fruits even more, the spider monkeys’ discrimination performance was slightly higher and statistically indistinguishable from their ability to discriminate between the full ripe and unripe odors (Fig.1, odor pair 2). Thus, we interpret all deviations from the baseline discrimination level (ripe vs. full unripe, odor pair 2) as statistical noise and are left to conclude that none of the manipulations of unripe odors caused any systematic decrease in the ability of the spider monkeys to discriminate between odors of ripe and unripe fruits. In summary, even as the odors of ripe and partially ripe fruits became increasingly similar, spider monkeys still readily discriminated between them and identified the full ripe odor mixture.

These results exemplify the acute sense of smell in spider monkeys and suggest that the ability to identify ripe fruits does not depend on any single compound. Further, these results show that even when the odors of ripe and unripe fruits become increasingly similar, the monkeys still readily discriminate between them and can use olfactory cues to determine whether a fruit is ripe or not. Finally, the results show that the monkeys quickly learn to successfully solve novel olfactory tasks (discrimination between a known rewarding and a variety of novel non-rewarding odors). This ability is beneficial because natural fruit odors are not uniform: individual fruits may develop under different conditions and therefore unripe fruits may emit some compounds in concentrations similar to the ripe fruits, and vice versa^15^. As a result, the ability to recognize the odor of ripe fruits against different combinations of partially ripe odor should allow spider monkeys to select fruits of an optimal degree of ripeness in a natural environment, in which signals are often accompanied with some degree of noise.

Thus, odor profiles of ripe *C. macrocarpa* and *L. cymosa*, which are composed of a plethora of different odorants, show a complexity that allows them to remain unique, and hence identifiable by spider monkeys and probably other primates and non-primate frugivores as well, even when the concentration of some compounds substantially deviates from the mean typical for a given ripeness level. This increases the signal’s specificity and ensures the reliability of communication despite inevitable noise with regards to the concentration of some compounds, and may therefore be an adapted feature of fruit odor in the two plant species. On the other hand, it should be considered that the biosynthetic machinery used for production of plant secondary metabolites is non-specific, and therefore volatile plant secondary metabolites are always produced in complex mixtures^24^. Therefore, it could be that if fruits are under selection to emit an odor that signals their ripeness, the only way to achieve that is through complex mixtures of volatiles. In this case, odor complexity of ripe *C. macrocarpa* and *L. cymosa* is an inevitable byproduct of signaling via the olfactory trajectory and it is possible that if a more compound-specific biosynthetic pathway for synthesis of volatile secondary metabolites were available, a simpler odor mixture could function equally well in conveying information to seed-dispersal vectors.

Our results further highlight that the sense of smell of a species cannot be summed up simply as “good” or “bad”. As fruit specialists that feed on fruits of many different plant species^25^, spider monkeys would benefit from the ability to learn olfactory discrimination tasks and maintaining high discrimination ability between complex mixtures even when signals include a substantial amount of noise. Other, more specialist species, may possess olfactory systems that serve them well in their respective ecological niche but which do not require to maintain such high discrimination capacity in diverse, noisy, conditions. So, their olfactory systems may be useful and good – but entail different capacities.

In conclusion, our study provides the first attempt to examine how primate olfactory discrimination capacity translates into success in ecologically realistic fruit-selection tasks. It confirms that spider monkeys achieve high discrimination performance between odor profiles of ripe and unripe fruits of two wild plant species, and therefore identify ripe fruits based on their volatile profiles. Further, our results show that the ability of spider monkeys to discriminate between the odors of ripe and unripe fruits does not depend on single compounds or compound classes. This unique odor signature, which retains information regarding fruit ripeness even when some noise is introduced, should enhance the overall attractiveness of the fruits to frugivores and therefore contribute to facilitating the mutually-beneficial interaction between plants and seed-dispersing primates and possibly other seed-dispersal vectors.

## Methods

### Animals

Testing was carried out using four adult female and one adult male black-handed spider monkeys *(Ateles geoffroyi)*. The male was 8 years old, and the females were 9, 10, 11, and 15 years old, respectively, at the start of the study. The spider monkeys were kept in outdoor enclosures at the UMA Hilda O’Farrill (environmental management unit), maintained by the Universidad Veracruzana near Catemaco, Veracruz, Mexico, and were thus exposed to natural environmental conditions concerning ambient temperature, relative humidity, and light. All spider monkeys had served as subjects in previous olfactory experiments and were familiar with the basic test procedure^26^,^27^,^28^. Maintenance of the animals has been described in detail elsewhere^11^. As they were all captive born, it is unlikely that they had been familiar with fruits of *Couma macrocarpa* and *Leonia cymosa* prior to the current experiments.

The experiments reported here comply with the *Guide for the Care and Use of Laboratory Animals* (National Institutes of Health Publication no. 86-23, revised 1985) and also with current Swedish, German, and Mexican laws. They were performed according to a protocol approved by the ethical board of the Federal Government of Mexico’s Secretariat of Environment and Natural Resources (SEMARNAT; Official permits no. 09/GS-2132/05/10).

### Odorants

Odor stimuli were synthetic odor mixtures mimicking the odors of ripe and unripe fruits of *Couma macrocarpa* (intact) and *Leonia cymosa* (intact and open) as well as of intermediate degrees of ripeness of both fruits. For this, we prepared mixtures mimicking the odor of partially-ripe fruits in which the concentration of one or more compounds as present in the unripe fruit was manipulated to match the concentration in the odor of the respective ripe fruit.

We used commercially available odorants (Supplementary Tab. S1 online) dissolved in near-odorless diethyl phthalate (99%, Sigma Aldrich, Germany). Although not all odor chemicals identified in the natural fruits (Nevo *et al.*) were available, we obtained most of the major components. This allowed reconstructing of a substantial proportion of the natural odors (*C. macrocarpa* - ripe intact: 84%, unripe intact: 84%; *L. cymosa* – ripe intact: 69%, unripe intact: 77%, ripe open: 87%, unripe open: 68%).

After mixture preparation we sampled their headspaces to verify that their odors resembled the odors of natural fruits. Sampling was conducted according to a protocol identical to the one used for analysis of natural fruit odor in Nevo *et al.* 1 ml of mixture was placed in an open 2 ml Eppendorf tube and placed inside a sealed chamber made from an unused inert baking bag (Toppits, Germany) for 2.5 h. The accumulated headspace was then collected for 10 min an a constant airflow if 330 ml/min onto a self-made absorbent trap containing 1.5 mg of Tenax-TA and 1.5 mg Carbotrap (both Supelco, Sigma-Aldrich, Germany). Absorbent traps were loaded at the tip of a cleaned Teflon tube which was the only opening in the system. Absorbent traps were loaded immediately afterwards to a Hewlett Packard HP 6890 Series gas chromatographic–mass selective detector (GC–MS; Agilent Quadrupol 5972) equipped with a DB-5ms capillary column (30 m long, 250 μm in diameter, film thickness: 0.25 μm, J&W) and analyzed in conditions identical to those described in Nevo *et al.* We then adjusted the concentrations of the odorants in accordance with the results, until the headspace was similar to the odor of natural fruits in both composition and intensity. To confirm that our synthetic mixtures sufficiently resembled the natural odors, we ran a principal component analysis (PCA) followed by a discriminant function analyses on the natural odor of ripe and unripe fruits of both species (data and analysis methods from Nevo *et al.*) and then verified that the synthetic mixtures scored on the DFAs similarly to the natural odors. All samples scored within the range of the natural odors on the discriminant functions and can thus be considered to be reasonable representatives of natural odors (supplementary Fig. S1, S2 online).

This procedure led to 6 basic “recipes” mimicking the full odors of ripe and unripe fruits of *C. macrocarpa* (intact only) and *L. cymosa* (intact and open) (supplementary Tab. S2, S3 online), which we also used to generate the partially ripe odors. Due to time and budget constraints, we did not test all possible combinations of compounds in partially ripe mixtures but focused on compounds that showed large differences between ripe and unripe fruits.

### Behavioral test

We assessed the olfactory discrimination performance of the spider monkeys using a food-rewarded two-choice instrumental conditioning paradigm^21^. The test apparatus consisted of a 50 cm long and 6 cm wide metal bar with two cube-shaped opaque PVC boxes with a side length of 5.5 cm attached to it at a distance of 22 cm from each other. Each container was equipped with a tightly closing hinged metallic lid, hanging 2 cm down the front of the container. From the center of the front part of the lid, a pin of 3 cm length extended towards the animal and served as a lever to open the lid. On top of each lid was a metal clip attached. This clip held a 70 × 10 mm absorbent paper strip (Schleicher & Schuell, Einbeck, Germany) which was impregnated at its distal end with 10 μl of an odorant used as rewarded stimulus (S+) or with 10 μl of an odorant used as unrewarded stimulus (S−). The paper strips extended approximately 3 cm into the cage when the apparatus was presented to the animals. The box with the absorbent paper strip bearing the S+ attached to the lid contained a food reward, a Kellogg’s Honey Loop®, while the one bearing the S− did not.

When presented with the test apparatus the monkeys sniffed both paper strips for as long as they liked and then decided to open one of the boxes. In the rare cases when a monkey tried to open a box without prior sniffing or tried to open both boxes, the experimenter held a chain connected to the lid tight so that the animal could not move the lid. After the decision and, in the case of a correct choice, after food retrieval the apparatus was immediately removed and prepared for the next presentation out of sight from the monkeys. Each monkey received three blocks of 10 trials (i.e., three sessions) per day. In five of the 10 trials of a session, the left box was baited and in the other five trials the right box was baited. The order of the “correct” and the “wrong” sides was pseudorandomized with the limitation that one box was not baited more often than three times in a row. At the end of each session the apparatus was thoroughly cleaned with 96% ethanol to ensure that no traces of odorants were left.

Control tests without a food reward being present in the box bearing the absorbent paper strip with the S+ resulted in the same high level of correct choices as tests with a food reward being present in the box. Further, previous studies have shown that the animals consistently failed to perform above chance level when the S+ was presented at subthreshold (i.e. undetectable) concentrations, despite a food reward being present in the box bearing the absorbent paper strip with the S+. Together, this excludes the possibility that the monkeys smelled the food reward inside the box or based their decisions on cues other than the odors of the S+ and the S−.

The animals were tested individually to avoid distraction from conspecifics. To this end, an animal voluntarily entered a small test cage (80 × 50 × 50 cm) adjacent to the group enclosure which could be closed by a sliding door for temporary separation. The animal sat on a bar mounted horizontally and parallel to the front side of the test cage. This front side of the test cage consisted of a stainless steel mesh with a width of 1 cm and had two openings of 5 × 5 cm allowing the animal to reach through the mesh, open the lid of one of the boxes of the test apparatus and to retrieve the food reward. The test apparatus could be attached to the outside of the front side of the test cage in such a way that the lids of the boxes were at a height consistent with the reach-through openings.

We assessed the ability to discriminate between the odors of ripe and unripe fruits, or between the odors of ripe and partially ripe fruits, by assigning one odor mixture mimicking the ripe fruit odor as the rewarded stimulus (S+), and several other odor mixtures representing different degrees of unripe fruit odor as the unrewarded stimulus (S−). In order to allow animals to build a robust association between a given odorant and its reward value, the critical tests started by assessing the ability to discriminate between a ripe fruit odor as S+ and the monomolecular odorant anethole (described by humans as smelling of aniseed) as S−.

With each stimulus combination, each spider monkey performed six sessions of 10 trials. The first three sessions were considered as training sessions intended to allow the animals to learn the differing reward values of the two stimuli, and the last three sessions were considered as critical sessions that were used for statistical analysis of discrimination performance. Data collection took place between May and September 2014. The spider monkeys were not maintained on a food deprivation schedule but were tested in the morning prior to the presentation of their daily ration of food.

### Experiments

#### Data analysis

For each individual animal, the percentage of correct choices from 30 trials per stimulus combination was calculated. Correct choices consisted both of animals opening a box equipped with the S+ and failing to open a box equipped with the S−. Conversely, errors consisted of animals opening a box equipped with the S− or failing to open a box equipped with the S+. Significance levels were determined by calculating binomial z-scores from the number of correct and false responses for each individual and condition. All tests were two-tailed and two different alpha levels were considerd: 0.05, corresponding to 21 out of 30 decisions (=70%) correct, and 0.01, corresponding to 23 out of 30 decisions (=76.7%) correct.

To assess whether discrimination performance within species/condition (e.g. *C. macrocarpa,* intact fruits) differed between treatments, we conducted a one-way non-parametric repeated-measures ANOVA (Friedman test). If the result of this test proved to be significant or approached significance, we further applied a post-hoc analysis of pairwise non-parametric repeated-measures Wilcoxon signed-rank tests, whose p-values were then subjected to the Bonferroni correction for multiple testing.

## Additional Information

**How to cite this article**: Nevo, O. *et al.* Chemical recognition of fruit ripeness in spider monkeys (*Ateles geoffroyi*). *Sci. Rep.* **5**, 14895; doi: 10.1038/srep14895 (2015).

## Supplementary Material

Supplementary Information

## Figures and Tables

**Table 1:** *Couma macrocarpa* – intact fruits.

	Full ripe vs.
1	Anethole
2	Full unripe odor
	***Partially ripe odors***
	*Terpenoids:*
3	Unripe + (*E-*) Caryophyllene
4	Unripe + α-Copaene
5	Unripe + monoterpenes (*E-*β-Ocimene, D-Limonene, Myrcene, Sabinene, γ-Terpinen)
6	Unripe + sesquiterpenes (α-Humulene, (*E-*) Caryophyllene, α-Copaene)
7	Unripe + monoterpenes + sesquiterpenes (*E-*β-Ocimene, D-Limonene, Myrcene, Sabinene, γ-Terpinen, α-Humulene, (*E-*) Caryophyllene, α-Copaene)
	*Aromatic compounds and aldehydes:*
8	Unripe + Ethyl salicylate
9	Unripe + Methyl salicylate
10	Unripe + aromatic compounds (Ethyl salicylate, Methyl salicylate, *p*-Cymene)
11	Unripe + *Trans*-2-nonenal
12	Unripe + aldehydes (*Trans*-2-nonenal + Nonanal)
13	Unripe + aromatic compounds + aldehydes (Ethyl salicylate, Methyl salicylate, *Trans*-2-nonenal, Nonanal)

**Table 2:** *Leonia cymosa* – intact fruits.

	Full ripe vs.
1	Anethole
2	Full unripe
3	Water

**Table 3:** *Leonia cymosa* – open fruits.

	Full ripe vs.
1	Anethole
2	Full unripe
	***Partially ripe odors***
	*Aromatic compounds:*
3	Unripe + Acetophenone
4	Unripe + Benzaldehyde
5	Unripe + *p*-Cymenene
6	Unripe + aromatic compounds (Acetophenone, Benzaldehyde, Cumene, *p*-Cymene, *p*-Cymenene)
	*Terpenoids:*
7	Unripe + α-Copaene
8	Unripe + *E-*β-Ocimene
9	Unripe + all terpenoids (*E-*β-Ocimene, α-Copaene)
	*Monoterpenes + aromatic compounds:*
10	Unripe + all aromatic compounds and monoterpenes (Acetophenone, Benzaldehyde, Cumene, *p*-Cymene, *p*-Cymenene, *E-*β-Ocimene)
