# Supplementary material for: Chemical recognition of fruit ripeness in spider monkeys (Ateles geoffroyi)
Source: Sci Rep. 2015 Oct 6;5:14895. doi: 10.1038/srep14895 (PMC4594300; doi:10.1038/srep14895)
Supplement: Supplementary Information [file srep14895-s1.pdf]

Nevo*, Orts Garri*, Hernandez Salazar, Schulz, Heymann, Ayasse & Laska

* Share first authorship

Chemical recognition of fruit ripeness in spider monkeys (*Ateles geoffroyi*)

Supplementary materials

Content:

- Tab. S1: origin and purity of compounds used for mixture preparations
- Tab. S2: absolute amounts of odorants in the synthetic mixtures of ripe and unripe intact *C. macrocarpa*
- Tab. S3: absolute amounts of odorants in the synthetic mixtures of ripe and unripe intact and open *L. cymosa*
- Fig. S1: Discriminant function analysis of intact *C. macrocarpa* fruits: natural odor and synthetic mixtures.
- Fig. S2: Discriminant function analysis of intact and open *L. cymosa* fruits: natural odor and synthetic mixtures.

Table S1: origin and purity of compounds used for all mixture preparations

**Common name**	**CAS**	**Purity**	**Origin**
Acetophenone	98-86-2	≥98%	Sigma Aldrich, Germany
Benzaldehyde	100-52-7	≥98%	Sigma Aldrich, Germany
(*E*-) Caryophyllene	87-44-5	80%	Dragon, China
Cumene	98-82-8	98%	Sigma Aldrich, Germany
Diethyl phthalate	84-66-2	≥99%	Sigma Aldrich, Germany
D-Limonene	5989-27-5	98%	Sigma Aldrich, Germany
Ethyl salicylate	118-61-6	99%	Sigma Aldrich, Germany
E-β-Ocimene	3779-61-1	90% + isomers	Dragon, China
α-Humulene	6753-98-6	96%	Sigma Aldrich, Germany
Linalool	78-70-6	97%	Sigma Aldrich, Germany
Methyl salicytate	119-36-8	99%	Sigma Aldrich, Germany
Myrcene	123-35-3	≥90%	Sigma Aldrich, Germany
Nonanal	124-19-6	≥95%	Sigma Aldrich, Germany
p-Cymenene	1195-32-0	≥98%	Sigma Aldrich, Germany
p-Cymene	99-87-6	99%	Sigma Aldrich, Germany
Sabinene	3387-41-5	75%	Sigma Aldrich, Germany
Trans-2-nonenal	18829-56-6	97%	Sigma Aldrich, Germany
α-Copaene	138874-68-7	70%	ACC Corporation, CA, US
γ-Terpinene	99-85-4	97%	Sigma Aldrich, Germany

Tab. S2: Recipe for odor mixtures mimicking the full odor of *C. macrocarpa* fruits. Amounts are in µl to create a mixture of 2ml. Odorant are from 100mg/ml solutions.

Odorant	Ripe intact (µl)	Unripe intact (µl)
(*E*-) Caryophyllene	94.92	28.57
α-Copaene	94.92	228.57
p-Cymene	0	1.14
Ethyl salicylate	20.34	0
α-Humulene	6.78	11.43
D-Limonene	0.20	5.71
Methyl salicytate	20.34	0
Myrcene	0.41	3.43
Nonanal	0.68	0
Trans-2-nonenal	6.78	0
E-β-Ocimene	2.71	0
Sabinene	0	2.29
γ-Terpinene	0.14	1.14
Solvent (diethyl phthalate)	1752	1718

Tab. S3: Recipe for odor mixtures mimicking the full odor of *L. cymosa* fruits. Amounts are in µl to create a mixture of 2ml. Odorant are from 100mg/ml solutions.

Odorant	Ripe intact (µl)	Unripe intact (µl)	Ripe open (µl)	Unripe open (µl)
Acetophenone	2.51	4.44	0	1.95
Benzaldehyde	2.82	2.54	9.76	6.35
α-Copaene	5.64	3.17	14.63	39.07
Cumene	0	1.59	0	0.32
p-Cymene	0.09	0.32	0	0.24
p-Cymenene	0	0	14.63	0.049
Myrcene	0	0	0.098	0.12
E-β-Ocimene	2.67	0	536.58	0.32
Z-β-Ocimene	0	4.44	0	0

Solvent (diethyl phthalate)	1986	1983	1424	1951

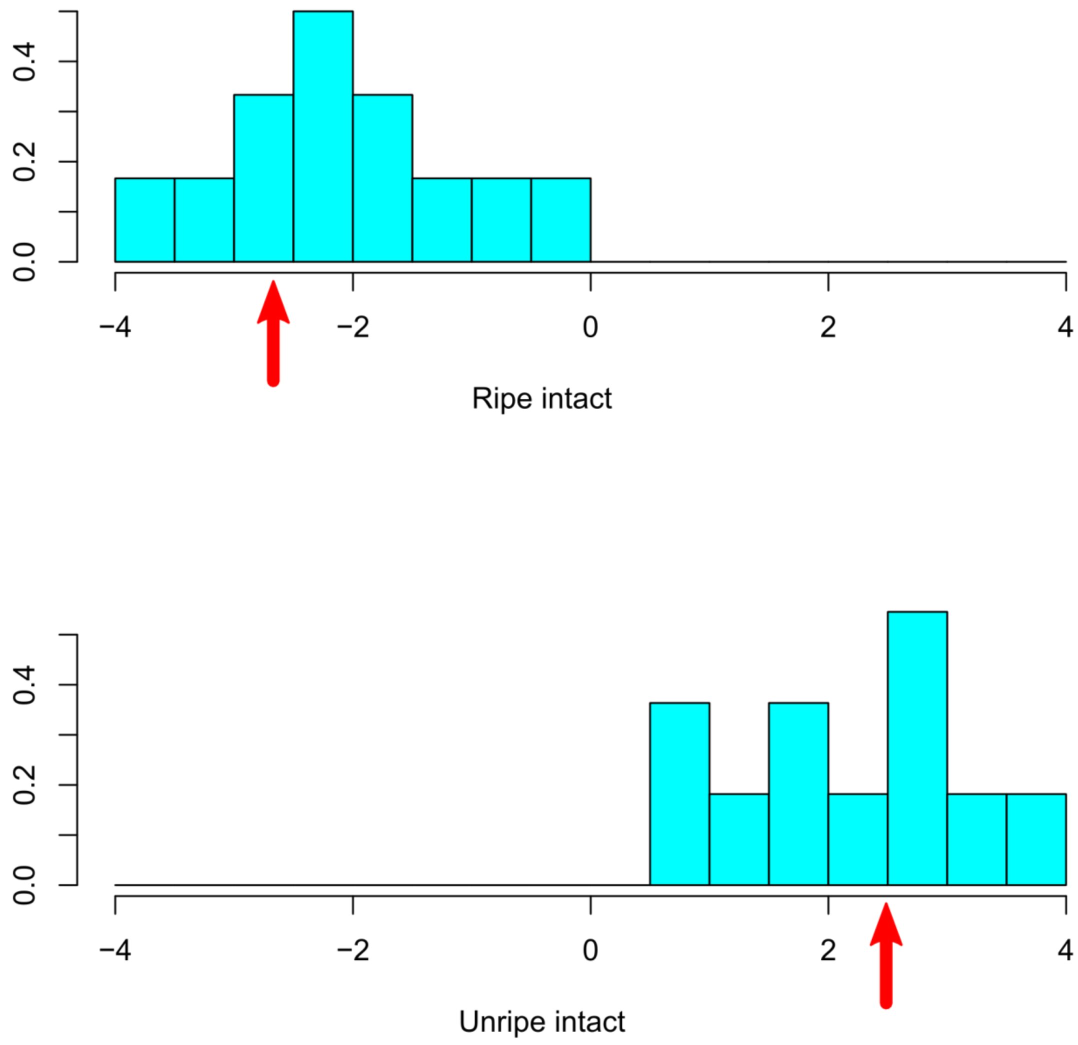

Fig. S1: Discriminant function analysis of odor of intact *C. macrocarpa* fruits. Data and analysis of natural fruit odor are identical to those in Nevo et al. Red arrows indicate the scores of the synthetic mixtures used in the bioassays in this study relative to their respective groups, and hence how representative they are.

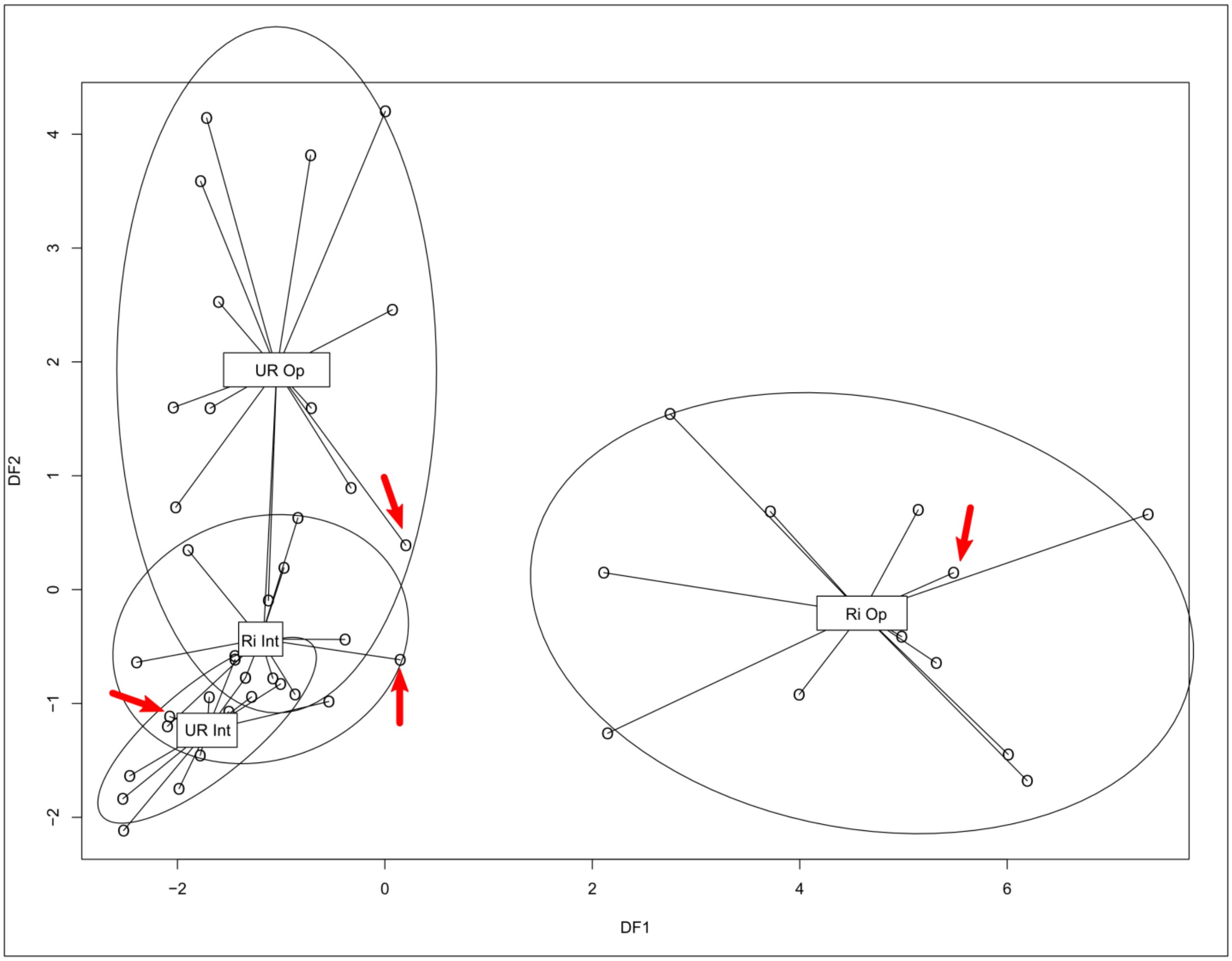

Fig. S2: Discriminant function analysis of odor of intact and open *L. cymosa* fruits. Data and analysis of natural fruit odor are identical to those in Nevo et al. Red arrows indicate the scores of the synthetic mixtures used in the bioassays in this study relative to their respective groups, and hence how representative they are. Ri Int: ripe intact; UR Int: unripe intact; Ri Op: ripe open; UR Op: unripe open.
